# Increased transcription of Glutathione S-transferases in acaricide exposed scabies mites

**DOI:** 10.1186/1756-3305-3-43

**Published:** 2010-05-18

**Authors:** Kate E Mounsey, Cielo J Pasay, Larry G Arlian, Marjorie S Morgan, Deborah C Holt, Bart J Currie, Shelley F Walton, James S McCarthy

**Affiliations:** 1 Infectious Diseases Division, Queensland Institute of Medical Research and Australian Centre for International and Tropical Health and Nutrition, University of Queensland, Brisbane, Queensland, Australia; 2 Tropical and Emerging Infectious Diseases Division, Menzies School of Health Research, Charles Darwin University, Darwin, Northern Territory, Australia; 3 Wright State University, Dayton, Ohio, USA; 4 Northern Territory Clinical School, Flinders University, Darwin, Northern Territory, Australia; 5 School of Health and Sports Science, University of Sunshine Coast, Maroochydore, Queensland, Australia

## Abstract

**Background:**

Recent evidence suggests that *Sarcoptes scabiei *var. *hominis *mites collected from scabies endemic communities in northern Australia show increasing tolerance to 5% permethrin and oral ivermectin. Previous findings have implicated detoxification pathways in developing resistance to these acaricides. We investigated the contribution of Glutathione S-transferase (GST) enzymes to permethrin and ivermectin tolerance in scabies mites using biochemical and molecular approaches.

**Results:**

Increased *in vitro *survival following permethrin exposure was observed in *S. scabiei *var. *hominis *compared to acaricide naïve mites (p < 0.0001). The addition of the GST inhibitor diethyl maleate restored *in vitro *permethrin susceptibility, confirming GST involvement in permethrin detoxification. Assay of GST enzymatic activity in mites demonstrated that *S. scabiei *var. *hominis *mites showed a two-fold increase in activity compared to naïve mites (p < 0.0001). Increased transcription of three different GST molecules was observed in permethrin resistant *S. scabiei *var. *canis*- mu 1 (p < 0.0001), delta 1 (p < 0.001), and delta 3 (p < 0.0001). mRNA levels of GST mu 1, delta 3 and P-glycoprotein also significantly increased in *S. scabiei *var. *hominis *mites collected from a recurrent crusted scabies patient over the course of ivermectin treatment.

**Conclusions:**

These findings provide further support for the hypothesis that increased drug metabolism and efflux mediate permethrin and ivermectin resistance in scabies mites and highlight the threat of emerging acaricide resistance to the treatment of scabies worldwide. This is one of the first attempts to define specific genes involved in GST mediated acaricide resistance at the transcriptional level, and the first application of such studies to *S. scabiei*, a historically challenging ectoparasite.

## Background

Scabies is a debilitating skin disease caused by the itch mite, *Sarcoptes scabiei*. It causes significant morbidity, especially in disadvantaged populations living in overcrowded conditions. In addition to the pathology directly attributable to scabies, it is a major initiating factor for streptococcal pyoderma, which in term is linked to renal and heart disease [1]. This association has led to concerted efforts to reduce prevalence of scabies and subsequent skin infections in settings where infestation is endemic, with studies demonstrating a link between scabies control and reduced incidence of post-streptococcal sequelae [2,3].

In northern Australia, the first line treatment for ordinary scabies is topical application of the pyrethroid acaricide 5% permethrin. Community control strategies have utilised permethrin extensively with varied success [4,7]. Limited sustainability of recent interventions [4] and anecdotal reports of treatment failure in some communities suggest the emergence of permethrin resistance as a possible cause for treatment failure. This is supported by observations that *S. scabiei *maintained on a laboratory animal model under permethrin selection developed resistance to this drug [8]. These sustainability concerns have now directed the development of alternative programs utilising the macrocyclic lactone drug ivermectin, with clinical trials targeting scabies and strongyloides in northern Australia due to commence in 2010 [2,9].

Despite widespread usage for sarcoptic mange in animals, ivermectin is a relatively new treatment for human scabies. It is the only oral acaricide available for scabies, and its most useful application has been in the management of hyperinfested (crusted) scabies [10] and in institutional settings. Ivermectin appears to have low residual activity against *S. scabiei*, and multiple treatments are required for severe crusted scabies. Recrudescence and re-infection occur frequently [reviewed in [11]]. Such treatment regimens inevitably impose selection pressure for drug resistance. Our group has documented clinical and *in vitro *ivermectin resistance in crusted scabies [12], as well as longitudinal evidence of increasing ivermectin tolerance in scabies mites collected from northern Australia [13]. Overall, these combined findings suggest the emergence of resistance to the two primary acaricides used in northern Australia. Defining molecular mechanisms of permethrin and ivermectin resistance in *S. scabiei *is therefore critical to future efforts to control this infection.

Possible mechanisms of acaricide resistance in *S. scabiei *may include 1) target site insensitivity and 2) increased drug efflux and/or metabolic detoxification. Studies to date suggest that both of these pathways are contributing to permethrin resistance in scabies. We have previously described a SNP in a *S. scabiei *voltage sensitive sodium channel gene (*Vssc*) associated with permethrin resistance [14], and likewise demonstrated increased esterase, glutathione transferase (GST) and cytochrome P450 monooxygenase activity in permethrin resistant mites compared to sensitive mites. Of these three metabolic pathways, GST appeared to be the most significant [8].

The development of ivermectin resistance in nematodes is apparently multifactorial, differing between organisms and perhaps even selection pressures [15]. The story is even less clear in arthropods, where there is very little molecular information regarding putative ivermectin resistance genes. Progress towards characterisation of ivermectin resistance associated molecules in *S. scabiei *has included the identification of several ABC transporter genes, including a P-glycoprotein [16] and a novel, ivermectin sensitive pH-gated chloride channel [17].

GSTs are a family of enzymes that play a significant role in detoxification of xenobiotics such as insecticides [18]. Increased activity of delta and epsilon class GSTs is linked to resistance to organophosphates, DDT and pyrethroids [reviewed in [19]]. GSTs have also been associated with macrocyclic lactone resistance in mites, with elevated GST activity observed in abamectin-resistant *Tetranychus urticae *[20,22]. Additionally, *Caenorhabditis elegans *isolates selected for ivermectin resistance *in vitro *show increased transcription of GSTs and glutathione conjugate MRP transporters, together with reduced intracellular glutathione, suggesting ivermectin induced acceleration of drug conjugation and removal [23].

The further characterisation of GSTs in *S. scabiei *is a logical step in exploring their possible role in mediating in resistance to both permethrin and ivermectin. To date, six GSTs have been identified from a *S. scabiei *expressed sequence tag library of 43,776 sequences. Three of these cluster with mu class GSTs, with the remaining three more related to the delta/epsilon classes of insects, which are of particular interest to drug resistance [24]. The objective of this study was to further investigate at a transcriptional level the role of GSTs and other putative resistance genes as mediators of acaricide resistance in *S. scabiei. *Transcriptional levels in different mite populations with different acaricide exposure histories and in different developmental stages were measured.

## Results

### GST inhibitors restore susceptibility of *S. scabiei *var. *hominis *to permethrin

**Figure 1:**
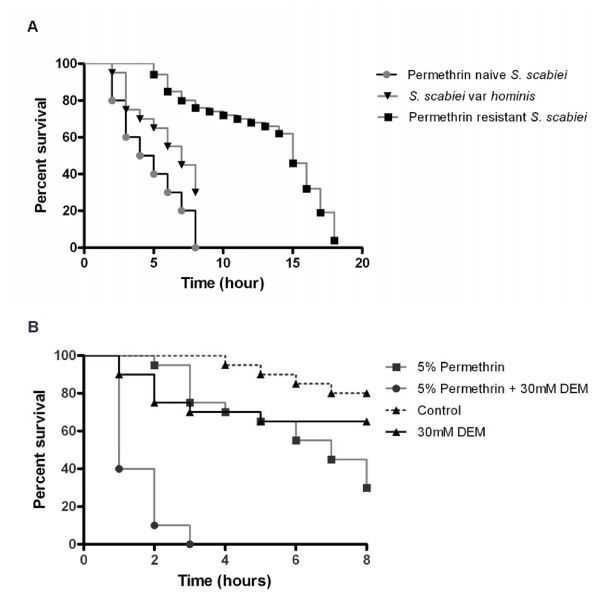
**In vitro bioassays of *S. scabiei *var. *hominis *permethrin sensitivity**. a) Differences in *in-vitro *permethrin susceptibility in *S. scabiei *with different acaricide exposure histories: Permethrin naïve *S. scabiei *var. *suis *(n = 100), exposed *S. scabiei *var. *hominis *(n = 40) and permethrin resistant *S. scabiei *var. *canis *(n = 100). Survival curves are significantly different (p < 0.0001). b) Synergistic activity of DEM. *S. scabiei *var. *hominis *mites exposed to permethrin combined with DEM (n = 40) show increased susceptibility compared to mites exposed to permethrin alone (n = 40) (p < 0.0001). Control mites exposed to mineral oil (n = 20) or DEM alone (n = 20) show little mortality over eight hours.

Mean survival time of *S. scabiei *var. *hominis *in the presence of 5% permethrin was 6 hours (95% CI 5.3-6.8). In contrast, previously published studies have reported a median survival time of 15 hours for permethrin resistant *S. scabiei*, and 4 hours for acaricide naïve *S. scabiei *[8]. Exploratory analysis of these different survival patterns was undertaken using Kaplan-Meier survival analysis, and indicated statistically significant differences in median survival times following permethrin exposure (p < 0.0001, Figure 1a). Therefore, these var. *hominis *mites were considered to be 'tolerant' to permethrin.

To investigate the relative contribution of GST metabolic pathways to increased permethrin tolerance, the synergistic compound and GST inhibitor DEM was also tested in bioassays. The addition of 30 mM DEM to 5% permethrin significantly reduced mean survival time of the var. *hominis *mites from 6 hours to 1.5 hours (95% CI 1.2-1.7, p < 0.0001, Figure 1b). Mites exposed to mineral oil 30 mM DEM alone exhibited little mortality (median survival > 8 hours), whereas mites exposed to the positive control acaricide benzyl benzoate were killed within one hour.

### Increased Glutathione S-transferase activity is associated with permethrin tolerance

**Figure 2:**
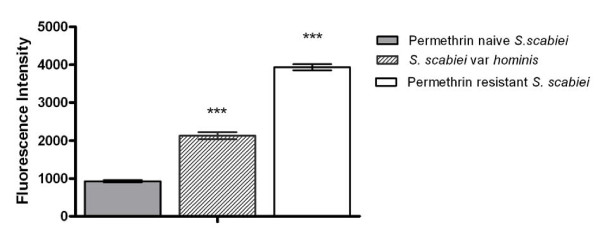
**Comparison of glutathione S-transferase enzymatic activity between mite populations**. GST activity was determined by a fluorometric assay measuring the conjugation of monochlorobimane to reduced glutathione. Permethrin resistant mites and human mites both display significantly (***, p < 0.0001) elevated GST activity compared to permethrin naïve mites. Bars represent median +/- SEM, n = 3 (protein extracts from female mites).

Given the increased *in vitro *permethrin tolerance and synergism by DEM observed in var. *hominis *mites in bioassays, we wanted to determine whether this phenotype was associated with increased GST enzymatic activity. Protein extracts obtained from *S. scabiei *var. *hominis *showed a two-fold increase in enzymatic activity compared to previously reported GST levels in acaricide naive *S. scabiei *(p < 0.0001). In contrast, GST activity levels in permethrin resistant mites were two-fold higher than that of the present var. *hominis *population, and four-fold higher than the permethrin naïve population [8] (Figure 2).

### Comparison of Glutathione S-transferases in different host-derived populations of *S. scabiei*

DNA sequence analysis was undertaken for all six GSTs in var. *suis, hominis *and *canis *variants of *S. scabiei*, and sequences deposited into GenBank (Accession no. GQ214687-GQ214698). All were ≥99% identical at the nucleotide level. Sequence identity was 100% at PCR primer binding sites and in regions selected for qPCR amplification. All SNPs were synonymous substitutions, with the exception of GST delta 1, where two non-synonymous SNPs were identified in the var. *canis *sequence resulting in a proline to serine substitution at residue 46, and a threonine to serine substitution at residue 157. However these sites were also polymorphic in the var. *hominis *and *suis *cDNA, with both SNPs represented. Therefore these appear to reflect different isoforms of GST delta 1 rather than host-related differences. Overall, this high level genetic identity indicated that that the genes were sufficiently similar to enable comparison of transcriptional levels.

### Increased transcription of GSTs in permethrin resistant *S. scabiei*

**Figure 3:**
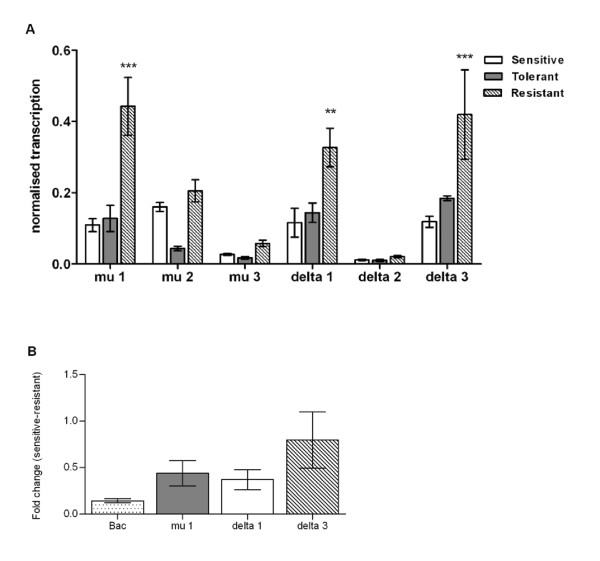
**Up-regulation of GST transcription in permethrin resistant *Sarcoptes scabiei***. Total RNA was extracted from pools of female mites (n = 4 pools per population) and reverse transcribed; GST transcripts were amplified using GST gene-specific primers in quantitative PCR, and normalised using levels of β-actin transcription. Bars represent mean +/- SE. ***: p < 0.001, **: p < 0.01 when compared to permethrin sensitive controls (b): Crude (non-normalised) fold-changes in expression in resistant compared to sensitive mites, showing little change in β-actin transcription between groups.

To investigate whether differences in GST activity corresponded with changes in transcriptional activity of GST genes, qRT-PCR was undertaken on representative *S. scabiei *GST genes. In the first set of analyses, transcription was compared between groups of female mites from permethrin naïve, permethrin resistant and permethrin tolerant mite populations. GST genes mu 1, mu 2, delta 1 and delta 3 were observed to be transcribed at high levels, whereas GST mu 3 and delta 2 transcripts were less abundant (Figure 3a).

We observed that GST mu 1, delta 1 and delta 3 were significantly upregulated in permethrin-resistant mites compared to permethrin-sensitive mites (Figure 3a). GST mu 1 transcription was increased by a mean of 4.2-fold (SE +/- 0.9, p < 0.001), GST delta 1 by 3.4-fold (SE +/- 0.9, p < 0.01) and GST delta 3 by 4-fold (SE +/- 1.79, p < 0.001). mRNA levels were slightly higher in the permethrin tolerant mites, compared to naïve mites, but these differences were not significant. Comparison of β-actin transcription between mite populations showed little variation between groups, indicating its suitability as a reference housekeeping gene that is constitutively transcribed (Figure 3b).

**Figure 4:**
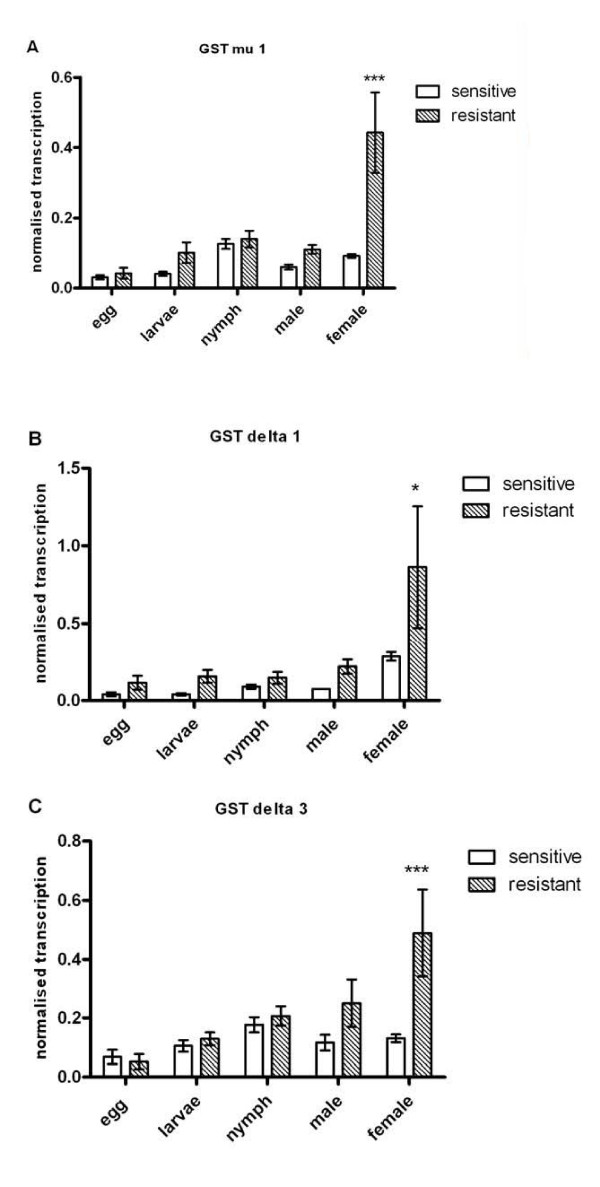
**Comparison of life-stage specific expression between permethrin sensitive and resistant *S. scabiei***. Levels of scabies mite GST transcripts from permethrin resistant and sensitive populations were compared across all life stages. GST mu 1 (a), delta 1 (b) and delta 3 (c) are expressed constitutively throughout the mite life-cycle. Resistant mites show increased mRNA at all life stages, with this trend reaching significance in female mites. *:<0.05, n = 3 pools per population.

Those GST isoenzyme genes whose transcription was up-regulated in permethrin resistant female mites were then examined in further detail, with transcriptional profiles compared across developmental stages in a single mite population. While transcripts of all three genes were present in all life stages, transcription was higher in resistant mites compared to sensitive mites all life stages, but was most marked and statistically significant only in female mites (Figure 4a-c).

### Increased GST transcription in mites clinically exposed to ivermectin

**Figure 5:**
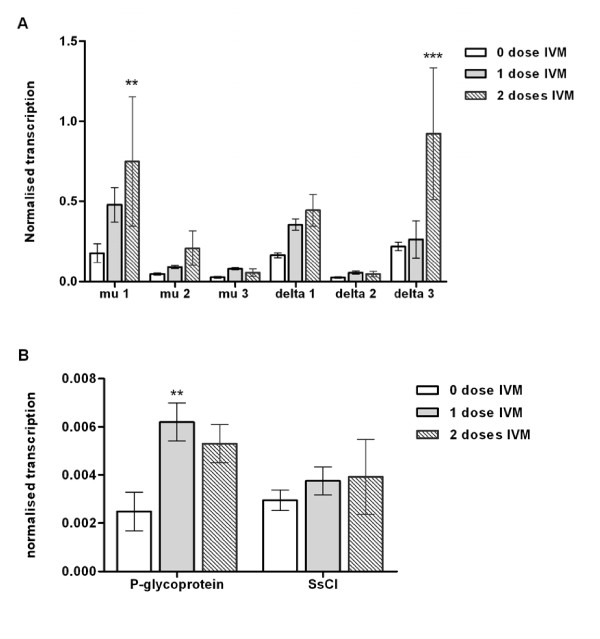
**Up-regulation of gene transcription in *S. scabiei *following clinical ivermectin exposure**. Changes in transcription of GST mu 1, GST delta 3 (a) and P-glycoprotein (b) in adult female mites exposed to ivermectin. Pools of female mites were collected from a crusted scabies patient prior to treatment (n = 4), after one dose of ivermectin (IVM, n = 4), and after two doses of IVM (n = 2). **:p < 0.01, ***:p < 0.001.

To investigate the effect of ivermectin treatment in a clinical setting on GST transcription, *S. scabiei *mites were collected from a crusted scabies patient before and after ivermectin treatment (Figure 5a). Transcription of GST mu 1 increased after one dose of ivermectin; after two doses this increase was significant (4.5-fold relative to untreated mites, SE +/- 3.4, p < 0.01). Significant up regulation of transcription of delta 3 was also observed after ivermectin exposure, but only after two doses (3.9-fold, SE +/- 0.82, p < 0.001). Although a trend towards increased GST delta 1 transcription was observed after one and two doses of ivermectin, this difference was not significant. Assay of transcription of a *S. scabiei *P-glycoprotein and pH-gated chloride channel indicated that P-glycoprotein transcription was increased by 2.9-fold after one dose of ivermectin (SE +/- 0.41, p = <0.01), while transcription of the pH-gated chloride channel (SsCl) was not affected by ivermectin treatment (Figure 5b).

## Discussion

Accumulating data indicate that metabolic detoxification plays an important role in the development of acaricide resistance. We have previously shown an association of GSTs with pyrethroid resistance in scabies mites, as determined by increased enzymatic activity and by the reversal of resistance using the GST specific inhibitor DEM [8]. We now extend on this by a) characterising the *in vitro *permethrin sensitivity and GST enzyme activity in *S. scabiei *collected from a patient with a history of extensive acaricide treatment, including permethrin, over ten years; b) confirming that the increased GST activity observed in permethrin resistant mites is correlated with up regulation of specific GST transcripts; and c) demonstrating that up regulation of GSTs and P-glycoprotein occurs in scabies mites over the course of ivermectin treatment.

Permethrin (5%) was introduced to northern Australia as a first line treatment for ordinary scabies in 1994. *In vitro *analysis conducted at that time demonstrated mite mortality within 30 minutes of permethrin exposure [25]. In later years, *in vitro *survival in permethrin had increased to 3 hours [26,27]. In the present study, median survival time had increased further to 7 hours. The addition of DEM restored permethrin susceptibility, in accordance with our previous data supporting GST mediated detoxification [8]. These phenotypic observations confirm increasing permethrin 'tolerance' in the var. *hominis *mites, but not complete resistance, as the resistant mite control population have a median survival time of 15 hours when exposed to permethrin [8]. This is consistent with the observation that GST enzymatic activity in the permethrin tolerant mites was two-fold greater than the unexposed controls, but two-fold lower than in the permethrin resistant mites.

Levels of transcription of GST in permethrin resistant *S. scabiei *were significantly increased relative to permethrin naïve controls. Up regulation was observed in transcript levels for three specific GST genes- mu 1, delta 1 and delta 3. Although the pattern of increased GST transcription in resistant mites was present across all life stages, it was most pronounced in adult females. GST levels in other mite species have been found to fluctuate dramatically as a function of adult female age, with this variation more pronounced in resistant mites [20,22]. Since the *in vitro *bioassays and enzymatic analysis in this study were conducted using only female mites, and in light of the transcriptional profiles, it would be interesting to also examine the drug sensitivity phenotypes of other developmental stages. If acaricide resistance is indeed only manifested in female mites, the inability to eradicate this stage would likely result in clinical treatment failure, since females are responsible for ongoing transmission of infection, and acaricides are not ovicidal.

The GSTs investigated in this study are likely to only represent a subset of the family of GSTs in the scabies mite. It is currently unknown how many members of this gene family are present in Acari, although this should become clearer as genome sequences become more readily available. Recombinant enzymes of these six GSTs have been expressed and kinetic properties determined. Docking analysis indicated that all but the GST mu 3 recombinant enzyme could bind and potentially metabolise permethrin [28]. Inhibition assays showed a 50% reduction of GST mu 1 activity in the presence of pyrethroid compounds [29]. To a lesser degree, permethrin also inhibited the activity of three recombinant GST delta enzymes [28].

Although the permethrin tolerant scabies mites showed significantly elevated GST activity relative to unexposed controls, increases in GST transcription were not statistically significant. This may be due to post-translational modifications or other factors exerting an influence on enzyme activity, or it may simply be that the level of tolerance is not yet high enough to be reflected at a transcriptional level. It is also important to acknowledge that in addition to possessing higher GST activity, the resistant mites carry a mutation in the permethrin target *Vssc *gene [14], a SNP not detected in the permethrin tolerant mites (data not shown). Hence, along with evidence implicating GSTs, it also appears plausible that *Vssc *SNPs, esterases, and to a lesser extent, cytochrome P450s may play a role in permethrin detoxification [8], as has been shown in other arthropods [30,32]. At this stage we cannot conclude whether our current observations represent a transient, isolated increase, or are part a trend of developing permethrin resistance in this region. However, the results suggest that permethrin treatment is having a distinct, albeit subtle effect on scabies mite populations in northern Australia.

Ivermectin has become a useful addition to the limited number of acaricides available for the treatment of scabies. However, there are legitimate concerns regarding the long-term efficacy of ivermectin as an acaricide due to observations of resistance in crusted scabies [12]. In this study, we assessed transcriptional levels of GSTs and other candidate ivermectin resistance genes in clinically obtained mites before and after ivermectin exposure. Significant up regulation of levels of transcription of GST mu 1, GST delta 3, and P-glycoprotein was observed in mites following treatment with one or two doses of ivermectin. This provides further support to an association with P-glycoprotein over expression and developing macrocyclic lactone resistance, as reported in ivermectin resistant *Haemonchus contortus *[33], emmamectin benzoate exposed *Lepeophtherius salmonis *[34] and in *C. elegans *selected for ivermectin resistance *in vitro *[23].

Developing hypotheses for mechanisms of GST mediated ivermectin resistance is difficult. The ivermectin molecule is considered too large to directly bind the GST active site, and there are no known glutathione conjugates of ivermectin. Nevertheless, evidence of a possible association between ivermectin and GSTs continues to accumulate. Studies of the recombinant *S. scabiei *GSTs showed that recombinant GST mu 1 activity was modestly inhibited by ivermectin (16%), and curiously, recombinant GST delta 3 activity was significantly *enhanced *by ivermectin (33%). While docking studies confirmed that ivermectin could not fill the active site of GST delta 3, it could bind the enzyme at several regions external to the active site. Thus it has been proposed that the enzyme may perform ligandin functions, sequestering ivermectin without metabolising it [28]. Increased transcription and sequestration could therefore reduce drug availability. Further crystallization and binding experiments are needed to investigate this hypothesis in more detail. An alternative hypothesis is that GSTs are indirectly involved in ivermectin detoxification, playing a secondary role to other known metabolic pathways such as cytochrome P450s.

Elucidating mechanisms of acaricide resistance in scabies mites is difficult due to the inability to maintain mites away from the animal host, and the sporadic access to patients with sufficient numbers of mites. Thus it is virtually impossible to conduct parallel phenotypic, biochemical and molecular studies due to the limited amount of material available. Although there were insufficient mites collected from this scabies patient to perform *in vitro *assays and biochemical profiling after treatment, an earlier study demonstrated selection for ivermectin tolerant sub-populations of mites over the course of treatment [13]. Here we have demonstrated that *in vitro *phenotypic changes in ivermectin response may be conferred at the transcriptional level. Given these current findings, caution should be exercised when using ivermectin alone, or in combination with permethrin due to the potential for cross resistance, mediated by GSTs as demonstrated in this study, or possibly by other metabolic mechanisms such as P450s.

## Conclusions

Despite the variant sources from which these results were obtained, the results advance the understanding of mechanisms of acaricide resistance in scabies. To date, most research on GST mediated acaricide resistance have been based on phenotypic response, use of synergists or biochemical assays, and the specific GST class or classes responsible have not been well defined. In this study the role for GST mediated acaricide resistance at the transcriptional level has been explored, and also the first report of P-glycoprotein up regulation in ivermectin exposed mites is presented. Altogether, our findings further validate multiple mechanisms of permethrin and ivermectin resistance in scabies mites. This trend of increasing tolerance to 5% permethrin, coupled with observations of emerging ivermectin resistance [12,13] raises serious concerns about the long term sustainability of current scabies treatments and highlights the need for development of alternative therapies.

## Methods

### Source of scabies mites

*Sarcoptes scabiei *var. *hominis *mites were collected from a patient with recurrent crusted scabies, admitted in November 2008 to Royal Darwin Hospital, Darwin, Australia. The patient resided in a remote community in northern Australia and had previous episodes of ivermectin treatment failure and resistance [12]. Mites were collected prior to treatment (Day 0), after one dose of ivermectin (Days 1 and 3) and after two doses of ivermectin (Day 7). Informed consent was obtained before mites were collected. This study was approved by the Human Research Ethics Committee of the Northern Territory Department of Health and Families and the Menzies School of Health Research.

*Sarcoptes scabiei *var. *canis *mites were originally collected from mange infested dogs and maintained on laboratory rabbit hosts, under permethrin treatment for many years [35]. These mites have a median survival time of 15 hours in permethrin [8], and thus were deemed to be "resistant" to permethrin. Rabbits were maintained in accordance with the institutional guidelines of the Wright State University Laboratory Animal Care and Use Committee.

*Sarcoptes scabiei *var. *suis *mites were harvested from a colony maintained on pigs in Brisbane, Australia. The mites obtained from this colony had no previous exposure to acaricides. *In vitro *survival time in permethrin was 4 hours [8]. Approval was obtained from the Animal Ethics committee of the Department of Primary Industry and Fisheries, Queensland.

### Bioassays of permethrin sensitivity in *S. scabiei *var. *hominis*

Mites were collected on Day 0, before any treatment was received. To circumvent reduced viability away from the host bioassays were initiated within 3 hours of mite collection. Bioassays were performed as described previously [8,27]. Mites were exposed to the following test compounds: a) 5% permethrin (n = 40); b) 5% permethrin + 30 mM diethyl maleate (DEM, Sigma, Milwaukee, WI, USA) (n = 40); and control compounds: c) 30 mM DEM (n = 20); d) mineral oil (negative control, n = 20); and e) 25% Benzyl Benzoate (positive control, n = 20). Mites were observed, and mortality recorded on an hourly basis for eight hours. Kaplan-Meier survival curves were constructed using Prism v5.0 (GraphPad Software, La Jolla CA). Comparison of survival curves was undertaken between the *S. scabiei *var. *hominis*, and previously conducted permethrin resistant (var. *canis*) and permethrin naïve (var. *suis*) [8] using logrank tests (GraphPad Prism).

### Assay of Glutathione S-transferase enzymatic activity

Approximately 100 female *S. scabiei *var. *hominis *mites were collected on Day 0 and homogenised on ice in 100 μL 0.05M Tris-HCl pH 7.5. The homogenate was centrifuged at 13,000 × *g *for 5 min at 4°C. Protein concentration of the supernatant was determined using the Nanodrop ND-1000 spectrophotometer (Nanodrop Technologies, Wilmington, DE, USA). GST activity was measured in a fluorometric assay using monochlorobimane (MCB) as described previously [8]. Each assay was performed in duplicate. Enzymatic activity of the *S. scabiei *var. *hominis *mites was compared to those previously determined for the permethrin naïve and resistant populations [8]. Statistical comparisons of mean activity between populations were made using Students *t*-test (GraphPad Prism).

### Analysis of levels of Glutathione S-transferase transcription

Transcription of GSTs was assessed in the context of different permethrin and ivermectin exposure histories. For permethrin, we compared permethrin "tolerant" *S. scabiei *var. *hominis *collected before treatment, permethrin naïve mites and mites known to be permethrin resistant. For ivermectin, mites were compared prior to treatment, and after one and two doses of ivermectin. As described above, mites were processed within 3 hours of collection to circumvent artefactual changes in trascription. Live *S. scabiei *mites were separated according to life stage (eggs, larvae, nymph, adult male and adult female) and stored in microfuge tubes in pools of 10-50 mites. *S. scabiei *var. *suis *and var. *hominis *mites were immediately homogenised in 50-100 μL cold TRIzol reagent (Invitrogen, Mount Waverly, VIC, Australia) and stored at -80°C until further processed. *S. scabiei *var. *canis *mites were stored in 100 μL RNA*later *(Applied Biosystems, Scoresby, VIC, Australia) for transport, then prior to processing the RNA*later *was decanted and mites homogenised in TRIzol.

Samples of pooled mites were thawed on ice and re-homogenised. After adding 400 μl TRIzol and 100 μL chloroform, the mixture was agitated and incubated at room temperature for 3 min. Samples were centrifuged at 10,000 × *g *for 15 min at 4°C, and the aqueous phase transferred to a chilled tube. Total RNA was purified and concentrated using MinElute RNA purification columns (Qiagen, Doncaster, VIC, Australia). RNA quantity and quality was measured using the Nanodrop ND-1000 spectrophotometer (Nanodrop Technologies, Wilmington, DE, USA) and Agilent BioAnalyzer (Agilent Technologies, Forest Hill, VIC, Australia) respectively.

One hundred nanograms of total RNA was reverse transcribed to cDNA using the Quantiscript RT kit (Qiagen). This kit uses a combination of random and oligo dT primers, and includes a pre-treatment genomic DNA removal step. Reverse Transcription reactions were incubated at 42°C for 30 min, followed by 95°C for 3 min. cDNA was diluted 1:1 in dH_2_O before using in PCR.

Levels of transcription of 6 GST genes as well as a P-glycoprotein and pH-gated chloride channel were analysed. The GSTs had been previously identified from scabies mite expressed sequence tag libraries and belong to the mu and delta classes [24] (Table 1). To exclude genetic divergence as a possible confounding factor in a comparative study of transcription, sequence analysis of GST genomic DNA and cDNA was undertaken from *S. scabiei *var. *suis*, var. *canis *and var. *hominis *isolates. Two mites from each variant host were compared. Each cDNA/gDNA was amplified using primers listed in Table 1 in a conventional PCR reaction; purified PCR products were sequenced using the Big Dye Terminator v3.1 sequencing kit (Applied Biosystems). The program ClustalW2 [36] was used to compare nucleotide and derived amino acid sequences between the different host-derived mite populations.

**Table 1:** Primers used in *Sarcoptes scabiei *qPCR and sequencing.

Gene	Accession number	Forward Primer (5'-3')	Reverse Primer (5'-3')	qPCR primer combination (product length)	Sequencing primer combination (product length)
**GST mu 1**	AF462190	F1: GCTATTGGGATCTTCGTGGA	R1:TGCCCAAATACCGGAGAATA	F1/R1 (228 bp)	F1/R2 (628 bp)
			R2: TGTATTCCATTTCGCCATTG		
**GST mu 2**	AY825933	F1: GCCCATCAGAATGATGCTTT	R1: TTCTCAAGATAATCTGGCTTTA	F1/R1 (344 bp)	F1/R2 (519 bp)
			R2: GAATCGATTAACATAGTTGCC		
**GST mu 3**	AY825934	F1: ATCTGGCGTGCAGATAAAC	R1: CTCGAGCCTTCTCGAAATTG	F1/R1 (241 bp)	F2/R2 (929 bp)
		F2: ACGCAGTTTTGTTTCGTTGG	R2: GTCTGGATTTGTTCCGTGGT		
**GST delta 1**	AY825935	F1: TGGACCAACATTAGCCGATA	R1: TTGCATTTGTTGAGCGAATC	F1/R1 (193 bp)	F2/R1 (631 bp)
		F2: CAGAAAGTGCACCATGTCGT			
**GST delta 2**	AY825936	F1: AGCTCAAACCGATGAGCCTA	R1:GCGAATGCAATGATGTTAGC	F1/R1 (192 bp)	F2/R1 (652 bp)
		F2:TGGGTTCTATTCGACCGATAA			
**GST delta 3**	AY825937	F1: ATGGAGGTGGTTTGAACGAG	R1: TCGTGATCGACAGCATTCAT	F1/R1 (244 bp)	F2/R1 (580 bp)
		F2: AGAGAGCCCACCATGTCGTA			
**P-glycoprotein**	DQ146410	AGGCAACTTCAGCACTCGAT	ACATTCTGACCGCCATCAAT	155 bp	
**SsCl**	EF611372	TGATTTCTATATGTCGGGCCATTTG	CAGGGAACCAAAGATCAACA	329 bp	
**β-actin**	EU624346	CAACCATCCTTCTTGGGTATG	CCAGCTTCGTCGTATTCTTGT	311 bp	

Quantitative PCR (qPCR) primers were selected on the basis of similar T_m _values and product length (Table 1). Primer sequences were queried with BLASTn to check that non-specific binding of human cDNA or that co-amplification of multiple genes did not occur. This was particularly important, as certain *S. scabiei *GST genes show relative sequence conservation [24]. To determine PCR efficiency, qPCR was performed on linearised plasmid cDNA clones for each of the genes investigated. Plasmid templates were quantified and serially diluted, with at least 5 dilutions used to construct standard curves. To confirm primer specificity and identity of amplified cDNAs, representative products from qPCR were subjected to DNA sequence analysis. Data was normalised to the reference housekeeping gene β-actin. This gene was selected due to its constitutive expression, lack of implication in drug resistance and its wide application in similar studies. To confirm its suitability for normalisation, levels of β-actin transcription were compared, and no significant variation was observed between life stages or population/drug exposure groups (Figure 2b).

Quantitative PCR was done using the QuantiTect SYBR green PCR kit (Qiagen). Reactions contained 1 X SYBR green master mix, 0.4 μM primers, 1 μL cDNA template and dH_2_O to a final volume of 10 μL. Reactions were cycled in the Corbett Rotor Gene 6000 real-time cycler (Corbett Research, Mortlake, NSW, Australia). Cycling conditions were: initial denaturation 95°C, 15 min, followed by 35 cycles of 94°C, 15 s; 56°C, 30 s; 72°C, 30 s; with data acquisition at 76°C, 20 s. Each reaction entailed amplification of the gene target in parallel with β-actin, allowing for normalisation. Each qPCR included an aliquot of a *S. scabiei *library cDNA [35,37] as a positive control and dH_2_O as a no template control. Each sample was also checked for genomic DNA contamination by testing a no-RT control using RNA as template. Ratios for relative transcription normalised to β-actin were calculated and groups compared using two way analysis of variance (ANOVA) with multiple comparisons using Bonferroni post-tests (GraphPad Prism). To estimate fold-change differences in transcription between populations, the efficiency corrected formula published by Pfaffl [38] was used.

## Competing interests

The authors declare that they have no competing interests.

## Authors' contributions

KM conceived and designed the study, collected samples, did bioassays, qRTPCR, sequence analysis, statistical analysis and interpretation, drafted the manuscript. CP participated in study conception and design, did bioassays, enzymatic assays, qRTPCR, sequencing, statistical analysis, drafted the manuscript. LA and MM facilitated sample collection and edited the manuscript. SW facilitated sample collection, participated in study design and interpretation, edited the manuscript. DH collected samples, did bioassays, edited the manuscript. BC facilitated sample collection, assisted with clinical interpretation, and edited the manuscript. JM participated in study conception and design, interpretation of data and helped draft and edit the manuscript.
